# Effects of Thermal Environment on Bone Microenvironment: A Narrative Review

**DOI:** 10.3390/ijms26083501

**Published:** 2025-04-09

**Authors:** Jiahao Yin, Qiao Guan, Minyou Chen, Yanting Cao, Jun Zou, Lingli Zhang

**Affiliations:** 1 College of Athletic Performance, Shanghai University of Sport, Shanghai 200438, China; 21210402@sus.edu.cn (J.Y.); 13659802234@163.com (M.C.); 15921372497@163.com (Y.C.); 2 School of Exercise and Health, Shanghai University of Sport, Shanghai 200438, China; 18325170695@163.com (Q.G.); zoujun777@126.com (J.Z.)

**Keywords:** thermal environment, bone metabolism, bone cells, bone microenvironment

## Abstract

Research findings reveal that thermal environments precisely regulate the skeletal system through a triple regulation of “structural morphology-cellular dynamics-molecular mechanisms”: At the tissue morphology level, moderate heat exposure can promote increased bone density and longitudinal growth, as well as improved fracture load and yield point, but may negatively affect geometric shape and cortical bone thickness. Continuous high-temperature exposure harms bone structure, manifested as changes in biomechanical characteristics such as decreased toughness and rigidity. At the cellular level, thermal environments directly affect the proliferation/apoptosis balance of osteoblasts and osteoclasts, and by regulating osteocyte network activity and bone marrow mesenchymal stem cell fate decisions, these four cell populations form temperature-dependent metabolic regulatory circuits. At the molecular dimension, heat stress can activate the release of neural factors such as CGRP and NPY, which possess dual regulatory functions promoting both bone formation and resorption; simultaneously achieving coordinated regulation of angiogenesis and fat inhibition through VEGF and TGFβ. The thermal environment–bone regulatory mechanisms revealed in this study have important translational value: they not only provide theoretical basis for biomechanical protection strategies for high-temperature workers and athletes, but also offer innovative entry points for analyzing the pathological mechanisms of heat stroke secondary bone injury and osteoporosis through heat stress-related signaling pathways, while establishing a theoretical foundation for the development of temperature-responsive functionalized biomaterials in bone tissue engineering.

## 1. Introduction

A thermal environment refers to an environment with a temperature or heat level that is higher than standard temperature conditions [1]. The increasing climate warming posed a serious threat to global health. In many countries, persistent high temperatures have become a serious issue and led to increased population mortality [2]. High-temperature environments can endanger specific populations, such as the elderly, individuals with cardiovascular diseases, pregnant women, children [3], outdoor workers [4], and athletes [5]. Biological organisms undergo three progressive stages in response to the thermal environment: heat exposure, heat stress, and heat shock response [6]. The three stages represent a continuous reaction process, from macro to micro and from the external environment to internal cells [7]. Heat exposure is the initial stage and occurs when an organism comes in contact with the thermal environment, which can lead to increased body temperature and potential risks [8]. As heat accumulates to a certain degree, the organism may enter the heat stress stage, which is characterized by overall discomfort [9]. The heat shock response is activated at the cellular level to cope with the potential damages caused by heat stress, which is a protective mechanism that involves the activation of complex signaling pathways [7]. The three stages form a progressively deepening continuum that reflects the adaptation and defense processes of biological organisms against the thermal environment, from the whole-body level to the cellular level.

The skeletal system, which is composed primarily of bone cells and the bone microenvironment, is a dynamic and complex system that renews and remodels continuously through the anabolic and catabolic processes of different bone cells to adapt to the changing environment [10]. Bone health is generally reflected in aspects such as the bone mass, bone biomechanics, and bone metabolism, and the homeostasis of the bone microenvironment reflects the dynamic balance within the skeletal system.

The impact of the thermal environment on bones has long been a subject of interest in sports and the life sciences. In 1877, Joel Allen first proposed the correlation between the thermal environment and bone length [11]. The field of thermal environment and bone research consistently presented a state of theoretical validation alongside academic controversy. Taking Allen’s rule as an example, Serrat (2013) confirmed a positive correlation between bone length and thermal environment through temperature intervention experiments [12], while Chevalier et al. (2020) found no significant correlation. This contradiction stems from differences in thermal environment parameters (such as intensity, duration, and intervention cycles) and their non-linear coupling with molecular regulatory networks [13]. Notably, a scholar ’s latest research (2024) provided cross-species evidence for Allen’s rule from an ecological evolutionary perspective, demonstrating correlations between climate warming and limb elongation in species such as masked shrews and Australian parrots [14]. Another scholar (2022) also found that Brazilian house mice have longer lower limbs than New York house mice, providing macroscopic evidence for Allen’s rule [15]. Recent studies revealed that temperature affects bones at multiple levels: Scandinavia has the highest osteoporosis rate, while Africa has the lowest [16]. The hip fracture incidence in Northern Europe is 11 times higher than in Mediterranean regions, suggesting that environmental temperature may exacerbate bone health risks [17]. Laboratory research progressed from initial bone morphology observations to molecular mechanisms of how heat exposure affects bone metabolism through indirect pathways such as gut microbiota and neural signaling. However, existing research has significant limitations, with different experiments employing vastly different temperature parameters (such as intensity, duration, and cycles), making results difficult to compare directly. This paper systematically examines the specific mechanisms of temperature effects on bone from three progressive levels: “bone morphology-cellular activity-molecular mechanisms”, establishing a clearer analytical framework for subsequent research.

## 2. Effects of Thermal Environment on Macroscopic Structure of Bones

### 2.1. Effects of Thermal Environment on Bone Morphology

The thermal environment can considerably influence the morphological structure of bones, including their length, thickness, and geometric shape. Previous introductions noted recent progress in validating Allen’s rule at the macro-ecological level: under climate warming, masked shrews experienced limb elongation, Australian parrots developed longer beaks [14], and Brazilian wild house mice have longer lower limbs than their New York counterparts of the same species [15]. More direct evidence comes from anthropological research—children in African populations exposed to monthly average temperatures above 35 °C show significant limb growth trends, and targeted heat interventions can non-invasively promote skeletal elongation [18]. Laboratory research, however, presents contradictions: some scholars found that mice raised at 27 °C had approximately 12% longer limbs than those in 7 °C environments, with significant growth in appendages such as ears and tails [19]. Research using localized heat exposure protocols (40 °C/40 min/7 days) similarly observed increases in tibia and femur length [20], yet another scholar using continuous exposure at 35 °C did not observe changes in bone morphology [13]. The key variable lies in the age of the experimental animals: the former used 3–4-week-old sexually immature mice, while the latter selected 8–16-week-old sexually mature individuals, indicating that temperature regulation of bone length has a clear developmental window effect [21].

The influence of the thermal environment on bone length may be attributed to multifaceted and comprehensive effects. First, heat exposure will affect the temperature of peripheral tissues, which in turn will influence the thickness of growth plates [22,23]. Growth plates play a crucial role in bone elongation [24]. The length of mice growth plates increases substantially in hot environments [22]. In addition to a direct effect of heat exposure, the increase in the growth plate length is related to leptin (LEP). LEP secretion by adipose factors increases significantly at a temperature of 41 °C [25]. LEP plays an important role in regulating bone growth, directly affecting growth plate chondrocytes, or promoting bone length increase by stimulating the hypothalamus to release growth hormones [26]. Second, heat exposure can cause vasodilation and thus alter blood flow and indirectly enhance the supply of nutrients and oxygen needed for bone growth and repair, which can promote bone length increase [22,23]. The thermal environment can also promote bone length increase by regulating transient receptor potential vanilloid 4 (TRPV4), which is an ion channel related to bone elongation [27]. TRPV4 can increase the cell volume of chondrocytes and thus promote bone elongation [28].

However, the thermal environment can have a negative impact on bone thickness. Offspring of pregnant mice under heat stress had thinner cortical bone compared with the control group [29]. Another scholar found that mice housed at 32 °C for 6 weeks developed thinner cortical bone [30], which may be related to the accelerated secretion of parathyroid hormone (PTH) in hot environments [31]. Continuous PTH expression will lead to an increase in the number of osteoclasts, which can lead to a reduction in cortical bone thickness [32].

The thermal environment can also induce changes in bone geometry. Humans who have been exposed to high-temperature environments for long periods may evolve narrow nostrils and skulls, which are highly effective for dissipating heat, to adapt to their environment [33]. Existing research has shown that mice housed at 32 °C for 6 weeks experience a decrease in total bone area [30], while another scholar’s research yielded similar results: heat exposure (43 °C, 60 min) can lead to the abnormal fusion of ribs and vertebrae in rats during their embryonic development, as well as malformations, such as a reduced number of vertebrae and ribs in the thoracic region [34]. Exposing pregnant rats to a hot environment of 42–42.5 °C yielded similar results. Examination of the embryos when the rats’ body temperature reached 42 °C revealed that the accelerated synthesis of heat shock proteins (HSPs) hindered the synthesis of the proteins necessary for normal embryonic development [35]. The expression of HSPs can be induced by various stressors, such as heat shock, and accelerated in hot environments [36], which will cause bones to miss critical development stages and ultimately lead to skeletal malformations [35].

In summary, existing research indicated that the thermal environment can exert a negative effect on bone geometry and thickness, although research on this topic is relatively scarce. Thus, further investigation into the effects of the thermal environment on bone morphology and structure is necessary to determine whether these negative effects are significant. However, the thermal environment can also have beneficial effects on bone length and density. Hot environments can directly regulate the growth rate of growth plates and thus promote bone length increase, which is a direct positive effect. In addition, hot environments can indirectly drive bone length growth by regulating thermosensitive hormones and related ion channels. Further research is needed to determine whether these positive effects are dominant over the negative impacts.

### 2.2. Effects of Thermal Environment on Bone Biomechanics

Core indicators of bone biomechanical properties include yield point, stiffness, fracture load, and toughness [37]. Research demonstrates that continuous heat exposure at 34 °C can significantly improve yield point (the critical stress value at which bone undergoes plastic deformation) and fracture load (the minimum force causing bone fracture) through regulation of gut microbiota [13]. The mechanism involves the restructuring of intestinal microbial communities: increased abundance of Akkermansia muciniphila, Bacteroides, and Alistipes promotes synthesis of polyamines (spermine/spermidine), while decreased Mucoraceae and Helicobacter inhibits polyamine degradation [13]. Elevated polyamine levels influence bone metabolism through dual pathways—on one hand, by downregulating cathepsin K (Ctsk), tartrate-resistant acid phosphatase 5b (Trap5b), and matrix metalloproteinase 9 (Mmp9) to inhibit osteoclast activity; on the other hand, by enhancing expression of osteocalcin (Ocn), osteoprotegerin (Opg), osteopontin (Opn), and alkaline phosphatase (ALP) to promote osteoblast function [13]. Notably, temperature threshold effects are significant: no biomechanical changes were observed after 6 weeks of intervention at 32 °C [30], while the reduction in stiffness, fracture load, and toughness required heat stimulation at 75 °C. Heating rat femurs to 75 °C for 60 s using microwave technology resulted in a 13% decrease in fracture load in three-point bending experiments [38]. Increasing the environmental temperature to 70 °C decreased the stiffness of rat femurs by 10%, while increasing the temperature further to 90 °C decreased the bone fracture load by 25% and stiffness by 33% [39]. Bone stiffness refers to the ability of bones to resist deformation under an external load [40]. This phenomenon may be attributed to the denaturation of collagen, as confirmed in an experiment where sheep femurs treated at 134 °C for 8 min exhibited decreased bone stiffness and fracture load. The bones’ internal collagen fiber structure changed, with the original collagen protein transforming into gelatin [41]. Collagen is a crucial nonmineralized tissue component that accounts for nearly half of the organic composition of bones and plays an important role in bone mechanics. The organizational morphology and cross-linking strength of collagen fibers can directly affect the inherent biomechanical characteristics of bones [42]. Rising temperatures can induce changes in the tertiary structure of collagen proteins, which can lead to collagen breakage [43]. In collagen molecules, some cross-linking involves thermally unstable aldimine bonds, which provide structural support for bones. The thermal environment can cause such bonds to break and recombine [44,45], which will result in damage to the overall collagen protein network and lead to reduced bone fracture load and bone toughness [43]. Bone toughness refers to the resistance and flexibility of a bone under fracture-causing forces [39]. However, some studies suggest that collagen denaturation may not lead to significant changes in bone stiffness [46]. The author argued that the factor that can affect changes in the bone stiffness is bone mineral density (BMD), which shows a noticeable change only when a bone is heated above 400 °C [47]. This discrepancy may be due to differences in research subjects (rat versus human femurs) or other mechanisms that have yet to be identified.

Therefore, exposure to higher temperatures (e.g., 75 °C) may reduce bone stiffness, fracture load, and toughness, potentially due to collagen denaturation. The extent to which collagen contributes to these changes remains debated, as other factors, such as differences in study subjects, may also play a role in the observed effects. However, direct heat exposure at 34 °C can enhance bone biomechanical properties, such as yield point and fracture load, through gut microbiota modulation.

### 2.3. Effects of Thermal Environment on Bone Mass

Bone mass refers to the bone tissue content and the bone matrix per unit volume, which are reflected mainly by the BMD and key factors that can lead to osteoporosis and fragility fractures [48]. Existing animal experiments thoroughly demonstrated the positive effects of heat exposure on bone mass, with both one-month exposure at 35 °C and six-month exposure at 32 °C enhancing bone formation and preventing bone loss [13,49]. Similar effects have been observed in humans as well. For example, high-temperature (100 °C) sauna bath tests on 23 adult males showed that muscle mass, bone mineral content, and BMD of their left leg increased significantly after heat exposure [50]. The potential mechanism of action is closely related to the effect of the thermal environment on bone metabolism. First, the thermal environment can promote the differentiation of bone marrow mesenchymal stem cells (BMSCs) and osteoprogenitor cells into osteoblasts and enhance bone mineralization. Second, heat shock can cause the upregulation of heat shock protein 70 (HSP70), and heat stress can upregulate the expression of vascular endothelial growth factor (VEGF), angiopoietin-1 (Ang-1), angiopoietin-2 (Ang-2), and tumor necrosis factorα (TNF-α), and thus further enhance bone formation.

**Figure 1:**
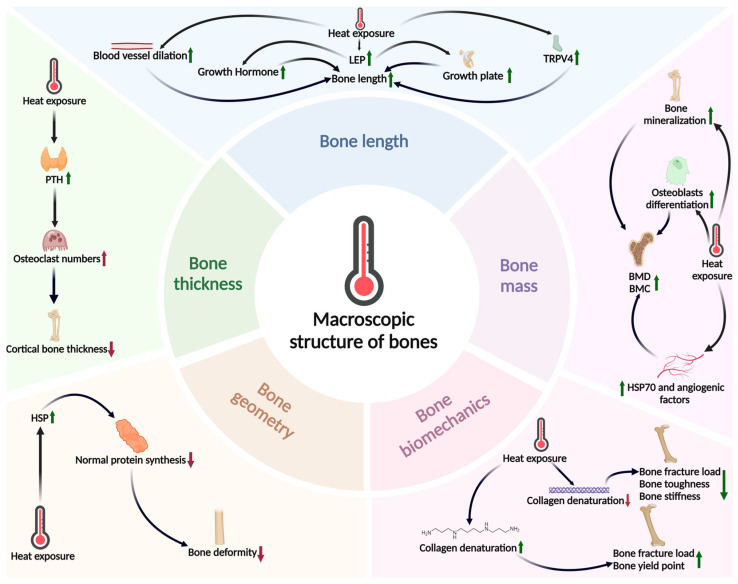
The thermal environment simultaneously influences bone length, bone mass, bone thickness, bone geometry, and bone biomechanical factors. This figure reveals that the effects of heat exposure on the skeletal system have multidimensional complexity: bone length growth is mainly related to angiogenesis and leptin signaling, but is age-limited (only significant during the bone growth period). The mechanism of bone thickness changes remains unclear, and although parathyroid hormone (PTH) can bidirectionally regulate osteoclast activity and bone formation processes, its net effect remains controversial. Changes in bone geometry may be related to heat shock proteins (HSPs) interfering with developmental protein functions, a process that also shows age dependency. At the biomechanical level, polyamines significantly improve bone yield points and fracture loads by enhancing osteoblast activity, while normal temperatures can avoid the negative effects of collagen denaturation. The mechanism of bone mass enhancement manifests as the synergistic effect of osteoblast proliferation and bone vascular proliferation, which can effectively increase bone mineral density (BMD) and bone mineral content (BMC). However, the apparent contradiction between increased bone mass and the cortical thinning phenomenon observed in some studies suggests that thermal environments may regulate bone quality and bone structure through different pathways, and the specific molecular mechanisms still need further verification. (Green indicates a positive effect on bone health, red indicates a negative effect, an upward arrow indicates upregulation, and a downward arrow indicates downregulation.).

However, the impact of the thermal environment on bone mass is not consistently positive. First, studies found that a single brief heat exposure event will not affect the BMD [51]. Second, as discussed previously, the thermal environment has significant negative effects on BMSCs and osteoblasts. However, current research on the direct impact of the thermal environment on bone mass is limited; thus, comprehensive and systematic clinical and basic research is necessary. (For detailed impacts of the above sections, please refer to Figure 1).

### 2.4. Effects of Thermal Environment on Bone Metabolism

Bone metabolism refers to the continuous cellular metabolic processes carried out by the bone cells in the human body. Bone metabolism is regulated mainly by two types of cells: osteoclasts and osteoblasts. The cells regulate bone remodeling through interactions such as direct contact. Bone metabolism balance disruptions may lead to diseases such as osteoporosis [52].

Numerous studies indicated that the thermal environment has a considerable promoting effect on bone formation. First, the thermal environment can directly affect BMSCs and osteoblasts through heat shock response and influence the osteogenesis process. Exposure to warm temperatures can increase the differentiation ability of BMSCs, promoting their differentiation into osteoblasts and facilitating bone formation [53]. Long-term exposure to temperatures within the range of 39–42.5 °C can induce cellular heat shock, which promotes osteoblast differentiation and enhances the formation of mineralized nodules [54]. Second, heat shock can upregulate HSP70 expression, which can directly affect bone formation. Heat stress can activate the p38 MAPK signal [55], and under heat stress conditions (43 °C, once daily, 15 min, 4 weeks), p38 MAPK pathway activation can induce Akt pathway activation, which can increase HSP70 expression [56]. HSP70 can enhance ALP activity, promote bone mineralization, and substantially increase the expression of osteogenesis-related genes, such as Runt-related transcription factor 2 (Runx2) and Osterix, and thus facilitate bone formation [57]. Moreover, the overexpression of heat shock 70-kDa protein 1A (HSPA1A), which encodes homologous HSP70, can enhance the osteogenic differentiation of BMSCs [36]. Last, mild heat stress can directly affect angiogenesis and osteogenesis, that is, angiogenesis and bone regeneration, by upregulating the expression of VEGF, Ang-1, Ang-2, and TNF-α [58].

The promoting effect of the thermal environment on bone resorption is the comprehensive result of multiple factors acting together. A thermal environment of 35 °C can reduce blood calcium ion concentrations and the intestines’ ability to absorb calcium [59]. When calcium ion concentrations decrease, PTH will promote the release of calcium ions from bones to maintain the calcium ion balance in the blood, thereby increasing bone resorption [59]. HSP70 and HSP90 from the HSP family can also indirectly affect bone resorption. HSP90 is a chaperone protein that can help other proteins fold correctly, protect proteins from heat stress, promote protein degradation, and be activated by the thermal environment [58]. However, the effect of HSP90 activation on bone resorption is controversial. First, HSP90 can inhibit osteoclast formation through Rab11a (a protease involved in vesicle transport) [60]. Second, as a chaperone of C-terminal Src kinase, the inhibition of HSP90 will activate Src kinase and thus promote osteoclast generation [36]. Inhibiting HSP90 with 17-AAG (an HSP90 inhibitor) accelerated osteoclast generation and promoted bone resorption [61]. However, inhibiting HSP90 with SNX-2112 (another selective HSP90 inhibitor) yielded contradictory results, inhibiting osteoclast formation and suggesting a reduction in bone resorption [62]. Therefore, the effect of HSP90 on bone resorption is complex and requires further discussion. However, the promoting effect of HSP70 on bone resorption has been confirmed; that is, it can promote bone resorption by inhibiting osteoclast apoptosis. HSP70 can protect partially folded structures and unfolded protein chains to inhibit aggregation, remodel folding pathways, and regulate activity [63]. HSP70 can also inhibit osteoclast apoptosis caused by the thermal environment by preventing the recruitment of procaspase-9 to apoptotic protease activating factor 1 (Apaf-1) and thus promote bone resorption [64].

**Figure 2:**
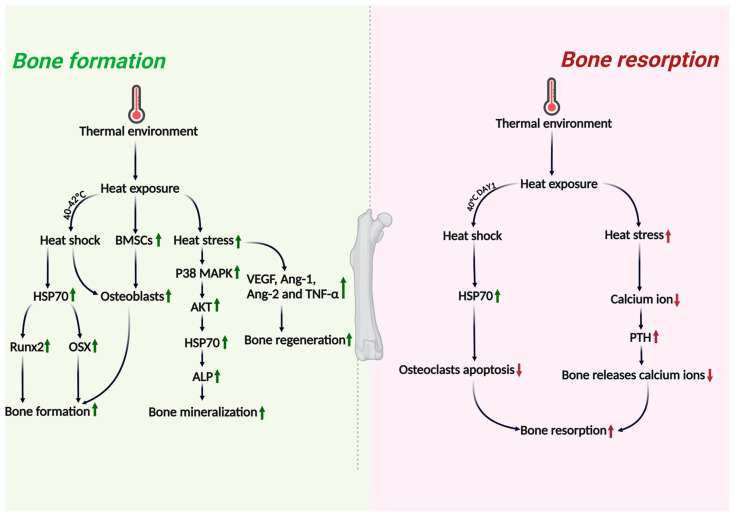
The differential effects of heat shock/heat stress on bone formation and bone resorption are illustrated. The diagram demonstrates distinct response patterns between these two fundamental bone remodeling processes when subjected to thermal stimulation. The impact of thermal environments on bone formation and bone resorption reveals that HSP70 appears to exert more positive effects on osteoblasts and positively influences bone formation through various factors and signaling pathways. In contrast, information regarding bone resorption is relatively scarce, seemingly limited to influences through anti-apoptotic effects and PTH. This indicates that the signaling pathways involved in thermal regulation of bone resorption still require further exploration. (Green indicates a positive effect on bone health, red indicates a negative effect, an upward arrow indicates upregulation, and a downward arrow indicates downregulation).

In summary, the effects of thermal environments on bone macrostructure are complex, manifesting not only at the molecular mechanism level, but also through numerous variables in experimental design. Beyond temperature, species and developmental age are also important factors that need to be considered. (For detailed impacts of the above sections, please refer to Figure 2, and for all the major experiments in this chapter, please see Table 1).

## 3. Effects of Thermal Environment on Bone Cells

### 3.1. Effects of Thermal Environment on BMSCs

BMSCs are mesenchymal stem cells and can be differentiated into different types of cells, namely, osteoblasts, adipocytes, chondrocytes, and endothelial-like cells [65].

Within a certain temperature threshold, the thermal environment can directly promote the osteogenic differentiation of BMSCs. However, beyond the threshold, the thermal environment can inhibit BMSC proliferation. Existing research confirms that long-term exposure to high temperatures (39 °C, 96 h, single exposure) can stimulate DNA synthesis in BMSCs, promote their differentiation into osteoblasts, and enhance BMSC mineralization [54]. However, exposure to high temperatures for extended periods (40–41 °C, 96 h, single exposure) or higher temperatures (42.5–45 °C, single exposure) for a brief period will inhibit BMSC proliferation [54]. Heat shock can enhance the expression of the HSPA1A gene in HSP70 [66], and high expression of HSPA1A can promote the differentiation of BMSCs into osteoblasts through the Wnt/β-catenin pathway, thus accelerating fracture healing. Therefore, heat shock induced by the thermal environment can promote the osteogenic differentiation of BMSCs and accelerate fracture healing [67].

Within a certain temperature threshold, the thermal environment can enhance the antiapoptotic ability of BMSCs and inhibit BMSC autophagy. Heat stress (42 °C, 60 min, single exposure) can substantially increase the expression of HSP27 and HSP70 in BMSCs [68], which play an important role in cellular antiapoptotic ability [69]. HSP27 can prevent the formation of the mitochondrial cytochrome c/Apaf-1/procaspase-9 apoptosome complex [70,71] and effectively inhibit the cleavage and activation of cysteine-aspartic protease-3 (caspase-3). Caspase-3 is an executor in the apoptosis process and can considerably enhance the intensity and duration of apoptotic signals [71,72]. HSP27 can also inhibit the release of the second mitochondria-derived activator of caspase (Smac) and reduce its binding to the X-linked inhibitor of apoptosis protein (XIAP). Smac is a protein that can promote cell apoptosis, and its binding to XIAP can also promote cell apoptosis. Therefore, HSP27 can enhance the antiapoptotic ability of BMSCs by inhibiting caspase-3 and Smac. HSP70 can inhibit cell apoptosis caused by the thermal environment by preventing the recruitment of the inactive precursor of caspase-9 (procaspase-9) to the Apaf-1 [64]. Mcginley [73] confirmed that HSP70 can improve the survival rate of BMSCs, reduce apoptosis, and maintain their multipotency [73]. In addition, HSP70 can prevent the transfer of the apoptosis-inducing factor to the cell nucleus and thus inhibit cell apoptosis [74,75]. The inhibition of BMSC autophagy through heat shock is one of the factors that can enhance cellular antiapoptotic ability. Wang [76] found that the apoptosis level of BMSCs was the lowest after 1 h in an environment with a temperature of 42 °C, and the expression of autophagy markers Beclin1 and microtubule-associated protein 1A/1B-light chain 3B was significantly reduced, which indicated that the thermal environment can inhibit cell autophagy [76].

The thermal environment can also alter the cellular structure of BMSCs. The thermal environment can increase the size and number of succinic dehydrogenase (SDH) in BMSCs, which is one of the main mitochondrial enzymes involved in cellular energy metabolism [54]. Previous studies revealed that biomineralization requires the participation of mitochondria, so the increased SDH activity will likely enhance the functionality of BMSCs [77]. However, sublethal heat shock conditions (45 °C, 30 min, single exposure) can cause BMSCs to develop premature senescence characteristics; that is, the cells will become large and flat [78].

**Figure 3:**
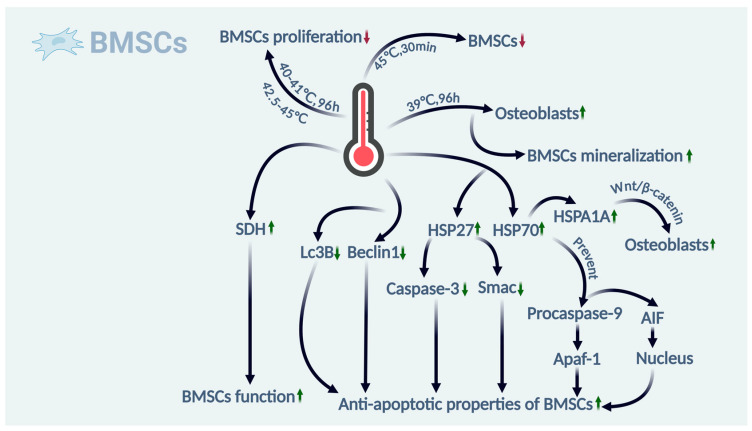
The effects of thermal environment on BMSCs. For BMSCs, moderate temperatures (39 °C) promote osteogenic differentiation, while high temperatures (40–45 °C) inhibit proliferation. The figure also shows that heat activates the Wnt/β-catenin pathway, thereby enhancing osteogenic differentiation in BMSCs, and it activates HSP27 and HSP70 to enhance anti-apoptotic capabilities. (Green indicates a positive effect on bone health, red indicates a negative effect, an upward arrow indicates upregulation, and a downward arrow indicates downregulation).

Overall, the effects of the thermal environment on BMSCs are complex. A certain temperature threshold can activate the Wnt/β-catenin pathway to promote osteogenic BMSC differentiation and increase BMSC antiapoptotic ability. The appropriate temperature can also increase the number of mitochondria in cells and inhibit BMSC autophagy. However, excessively high temperatures can inhibit BMSC proliferation, cause changes in the cellular structure, and lead to premature cellular senescence. (For detailed impacts of the above sections, please refer to Figure 3).

### 3.2. Effects of Thermal Environment on Osteoblasts

The effects of the thermal environment on osteoblasts are multifaceted.

From the perspective of the cellular structure, heat shock from exposure to temperatures below 45 °C for less than 10 min can lead to the destruction of the actin cytoskeleton but substantially increase the number and volume of mitochondria in osteoblasts [79]. Heat shock from exposure to an environment with a temperature of 40 °C for 4 days will result in a significant increase in osteoblast volume and protein content, as well as enhanced mitochondrial activity [80]. However, severe heat shock from exposure to an environment with a temperature of 47 °C for 1 min will cause cell membrane protrusions, cell detachment, and rounded cell bodies, which are characteristics of cell necrosis and apoptosis [54].

From a functional perspective, heat shock from exposure to certain temperatures (40–42 °C) can increase osteoblast metabolic activity and cell proliferation and promote the secretion of bone-related proteins by osteoblasts, such as OCN, osteopontin, and OPG, and thus bone regeneration without causing cellular toxicity. Osteoblast water bath treatment at 39–41 °C for 1 h can promote their proliferation and ALP production and induce the high expression of HSP27, HSP47, and HSP70. HSP27 and HSP70 have a positive effect on osteoblast survival and functions [81]. As mentioned previously, mild heat stress can increase VEGF expression [58]. VEGF can directly act on osteoblasts, promoting their proliferation, migration, and differentiation [82]. The thermal environment may also affect bone growth and repair by promoting the release and increase in growth factors, such as transforming growth factor-β (TGF-β) [83]. TGF-β can regulate osteoblast proliferation and differentiation [84]. Furthermore, the thermal environment can activate the Wnt signaling pathway under specific conditions [85], which is one of the key natural signaling pathways that can affect bone formation. The activation of the Wnt signaling pathway can actively promote osteoblast differentiation and functions [86]. This pathway can be activated at temperatures between 44.8 °C and 46.6 °C, but inhibited when the temperature exceeds 47.5 °C [85]. As mentioned previously, PTH secretion increases in a thermal environment. PTH can activate the IGF signaling pathway and promote aerobic glycolysis and mitochondrial respiration in osteoblasts and thus enhance their glucose utilization and lactate production in vitro [87]. PTH can also activate the BMP signaling pathway in osteoblasts and enhance the phosphorylation of SMAD family member 1 and SMAD5 proteins. Endogenous PTH can promote the expression of BMP receptor 2 in osteoblasts through the cAMP-PKA-CREB signaling pathway and Runx2 expression through the BMP-pSMAD1/5/8 signaling pathway and hence promote bone formation [88,89] and inhibit the expression of the Dickkopf-1 protein (a natural Wnt antagonist) and activate the Wnt signaling pathway to promote bone formation [90].

However, the effects of the thermal environment on osteoblasts also have negative aspects. Studies demonstrated that PTH can activate the Notch signaling pathway in osteoblasts by inducing increased expression of the Notch ligand Jagged1. As Jagged1 acts as a negative regulator in the osteogenic process, this activation can inhibit bone formation [90]. Furthermore, osteoblast growth stagnates after 96 h of exposure to a temperature of 39 °C (single exposure) [80] and suppresses proliferation. [54]. The thermal environment can also lead to decreased cell viability, necrosis, and apoptosis, which are closely related to temperature. Specifically, exposure to temperatures above 45 °C for 1 min can substantially reduce cell viability, whereas a temperature of 47 °C can increase cell necrosis and apoptosis [91].

**Figure 4:**
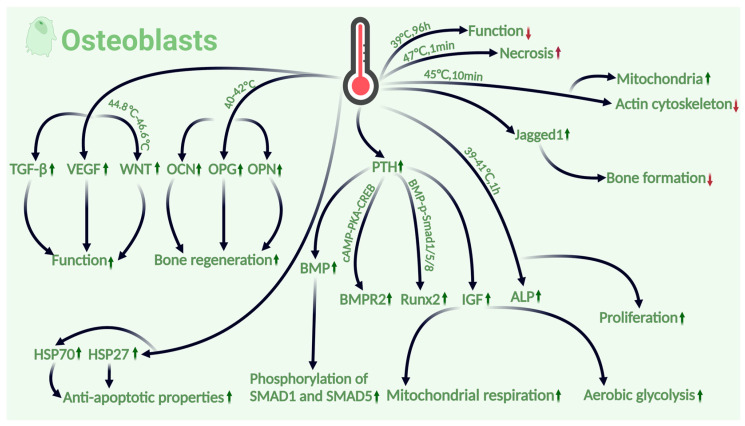
The effects of thermal environment on osteoblast. The osteoblast section demonstrates how moderate temperatures (39–42 °C) increase metabolic activity and proliferation, while high temperatures (>45 °C) reduce viability and increase apoptosis. Thermal environments also activate multiple signaling pathways (Wnt, IGF, and BMP) and increase VEGF, TGF-β, and PTH expression to enhance osteoblast function. (Green indicates a positive effect on bone health, red indicates a negative effect, an upward arrow indicates upregulation, and a downward arrow indicates downregulation).

In conclusion, the temperature and exposure duration are key factors that can affect the impact of the thermal environment on osteoblasts. Exposure to a moderate thermal environment (39–42 °C) for short periods will have a promoting effect on the osteoblast structure, functional metabolism, and proliferation. However, prolonged exposure or excessively high temperatures (above 45 °C) can lead to osteoblast growth stagnation, considerable downregulation of their activity, and obvious cell necrosis and apoptosis. (For detailed impacts of the above sections, please refer to Figure 4).

### 3.3. Effects of Thermal Environment on Osteocytes

Osteocytes play a crucial role in bone remodeling and can recruit nearby osteoclasts and osteoblasts to regulate bone resorption and formation and thus respond to mechanical stimuli and physical damage [92]. Osteocytes are more resistant to heat-induced apoptosis than osteoblasts and play a commanding and regulatory role in sensing and triggering the bone remodeling process caused by thermal damage [91]. After their exposure to the thermal environment (47 °C, 1 min), osteocytes will send signals to respond to the thermal damage. The apoptotic osteocytes will signal to the osteoblasts, whereas the healthy osteocytes will initially signal to the osteoclasts. The osteocytes will produce a receptor activator of nuclear factor kappa-B ligand (RANKL) to stimulate osteoclast differentiation and subsequently signal to the osteoblasts. In addition, the osteocytes will produce OPG and cyclooxygenase-2 to regulate the bone remodeling [91].

From a cellular structural perspective, thermal damage induces significant shrinkage and condensation of the cytoskeleton and cell membranes in osteocytes, leading to detachment from the matrix, a rounded shape, and ultimately, an unhealthy, near-death state [91]. From a cellular functional perspective, the thermal environment can induce osteocytes to send remodeling signals, and thus plays a crucial role in osteoclast activity, cell necrosis, and apoptosis. As mentioned previously, HSP27 and HSP70 activated by the thermal environment will have protective mechanisms against cell apoptosis. However, exposure of osteocytes to 45 °C for 1 min increases cell apoptosis, whereas raising the temperature to 47 °C for 1 min significantly reduces osteoclast activity and accelerates cell death. In conclusion, high temperatures in the thermal environment lead to decreased osteocyte viability, apoptosis, and death, but lower temperatures allow osteocytes to regulate bone remodeling.

### 3.4. Effects of Thermal Environment on Osteoclasts

Osteoclasts, multinucleated cells responsible for bone resorption [93], are influenced by the thermal environment in a complex manner. On one hand, high-temperature treatment (47 °C for 1, 3, and 7 days) has been shown to substantially decrease RANKL expression in osteocytes, leading to a corresponding decrease in the RANKL/OPG ratio, an important indicator of osteoclast activity [91]. On the other hand, osteocyte apoptosis can stimulate surrounding healthy osteocytes to produce the pro-osteoclast factor RANKL [94], potentially counteracting the high-temperature-induced decrease in RANKL expression. Adding to this complexity, HSP27 and HSP70, when activated by the thermal environment, can enhance the antiapoptotic ability of osteoclasts [81]. However, despite these regulatory mechanisms, the direct impact of high temperatures on osteocyte apoptosis remains significant and cannot be ignored [95].

The activation of the growth factor TGF-β by the thermal environment is another factor that can affect osteoclasts [83]. It can directly inhibit the proliferation and differentiation of osteoclast precursor cells [96].

**Figure 5:**
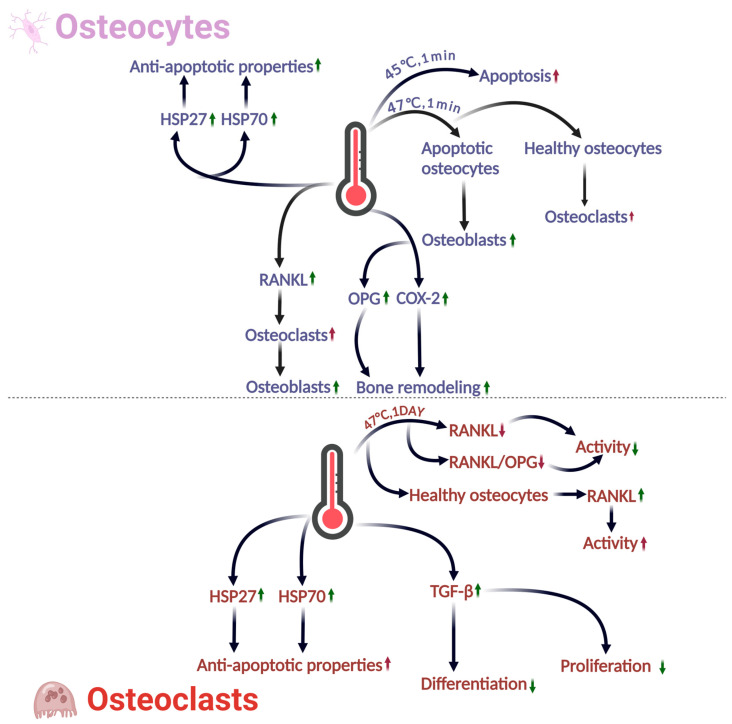
The effects of thermal environment on osteocytes and osteoclasts. Osteocytes are more heat-resistant than osteoblasts and regulate bone remodeling post-thermal injury. The figure shows them signaling to osteoblasts and osteoclasts, producing OPG and COX-2, but increasing apoptosis and cell death at 45 °C. Finally, the osteoclast section explains that high temperatures reduce RANKL expression and the RANKL/OPG ratio, and heat-activated TGF-β inhibits the proliferation and differentiation of osteoclast precursor cells, but osteocytes increase RANKL expression, demonstrating the complex effects of thermal environments on osteoclasts. (Green indicates a positive effect on bone health, red indicates a negative effect, an upward arrow indicates upregulation, and a downward arrow indicates downregulation).

In summary, the effects of the thermal environment on osteoclasts are complex. On the one hand, the thermal environment can enhance the antiapoptotic ability of osteoclasts and indirectly upregulate osteoclast activity by influencing osteocytes. On the other hand, excessively high temperatures can lead to rapid cell apoptosis and indirectly inhibit the development, proliferation, and differentiation of osteoclasts by downregulating osteocyte-related factors and activating growth factors. (For detailed impacts of the above sections, please refer to Figure 5, and for all the major experiments in this chapter, please see Table 2).

## 4. Effects of Thermal Environment on Bone Microenvironment

### 4.1. Effects of Thermal Environment on Bone Nerves

Bone nerves are crucial to skeletal development and repair and can regulate bone growth and regeneration by releasing signaling molecules, such as neurotransmitters, neuropeptides, and neurotrophic factors. The factors can regulate the functions of the nervous system and considerably impact bone metabolism and thus form an important link between the nervous system and the skeleton. The thermal environment may regulate bone health by affecting the release of the aforementioned signaling molecules. Moreover, the thermal environment can substantially upregulate various factors in bone nerves, including norepinephrine (NE), calcitonin gene-related peptide (CGRP), substance P (SP), neuropeptide Y (NPY), vasoactive intestinal peptide (VIP), nerve growth factor (NGF), and brain-derived neurotrophic factor (BDNF), which can influence bone metabolism and the bone microenvironment in multiple ways.

NE is one of the most crucial neurotransmitters in the sympathetic nervous system within bones. It has been observed that heat stress stimulation at 39 °C increases NE concentrations [97]. NE can activate β-adrenergic receptors (β-AR) on the surface of osteoblasts and thus inhibit bone formation. β-AR activation can also induce osteoblasts to produce interleukin-6 (IL-6) and IL-11, which are interleukins that can stimulate osteoclast differentiation [98]. Furthermore, β-AR can mediate the production of intracellular reactive oxygen species and thus directly regulate osteoclast generation [99]. However, it can also stimulate osteocytes to produce RANKL and hence promote osteoclast differentiation [100]. Therefore, NE can considerably stimulate bone resorption.

The thermal environment can also regulate bone metabolism by influencing CGRP, which is an important neuropeptide. Specifically, heat exposure at 47 °C has been found to activate the heat-sensitive ion channel TRPV1, leading to a substantial release of CGRP [101]. CGRP plays a regulatory role in communication between immune cells and osteoblasts, and thus can inhibit bone resorption [102]. It also plays a role in OPG/RANKL signal transduction by inhibiting osteoclast activation and bone resorption processes [103]. In addition, BMSCs under CGRP influence show significant improvements in proliferation, recruitment to ossification sites, and osteogenic differentiation, with enhanced ALP and Runx2 expression. CGRP can also promote the osteogenic differentiation of BMSCs by activating the Wnt/β-catenin signaling pathway [104] and stimulate osteoblast differentiation by upregulating the expression of activating transcription factor 4 (ATF4) and OCN [103]. The results indicate that the thermal environment can promote bone formation and inhibit bone resorption by regulating CGRP and its downstream signaling pathways. Another neuropeptide that can be activated by the thermal environment is SP, which is similar to CGRP. Heat exposure can activate TRPV1 and lead to the release of SP [105]. SP can promote bone formation by increasing cAMP generation, promoting osteoblast differentiation, and enhancing the secretion of bone morphogenetic protein-2 (BMP-2) [106]. However, studies revealed that SP can stimulate bone resorption by activating neurokinin-1 expressed on osteoclasts [107]. The synergistic effect of SP and CGRP is highly complex. In vitro experiments showed that SP and CGRP can individually enhance BMP-2 signaling and mineralization; however, when SP and CGRP jointly stimulate osteoblasts, they will downregulate the BMP-2-induced osteogenic differentiation [108]. Therefore, the mechanisms through which SP and CGRP affect bones are complex and diverse and require further in-depth research. It has been shown that heat exposure at 40 °C for 2–5 h can increase NPY expression [109]. NPY can affect bone metabolism through central and peripheral pathways, and previous studies clearly demonstrated that NPY can inhibit bone formation [110,111]. Meanwhile, exposure to 41 °C heat significantly elevates VIP expression [112]. VIP can activate functional receptors in osteoblasts, promote the osteogenic differentiation of bone cells, stimulate angiogenesis, increase VEGF expression in BMSCs, promote cAMP production, and downregulate osteoclast generation [110,111,113]. In summary, in the thermal environment, CGRP can promote bone formation while inhibiting bone resorption, and SP can also promote bone formation. However, when CGRP and SP act together, their bone formation-promoting effect will be downregulated. Furthermore, NPY can inhibit bone formation, whereas VIP can promote it.

The thermal environment can also activate NGF and BDNF. It has been shown that heat exposure at 37 °C enhances TRPV3 channel activity, leading to increased NGF expression [114]. The neurotrophin receptors of NGF, that is, tropomyosin receptor kinase A (TrkA) and p75 neurotrophin receptor (p75NTR), play a crucial role in osteoblasts and osteoclasts. NGF can prevent osteoblast apoptosis and promote osteoblast growth and differentiation [115]; however, it can also induce osteoclast generation independently without relying on RANKL [116]. Similar to NGF, heat exposure at 42 °C has been observed to increase BDNF expression [117]. BDNF can promote osteoblast growth and differentiation [118] and induce BMSCs to secrete RANKL, which will promote osteoclast formation [119]. In conclusion, NGF and BDNF have significant promoting effects on bone formation and bone resorption.

**Figure 6:**
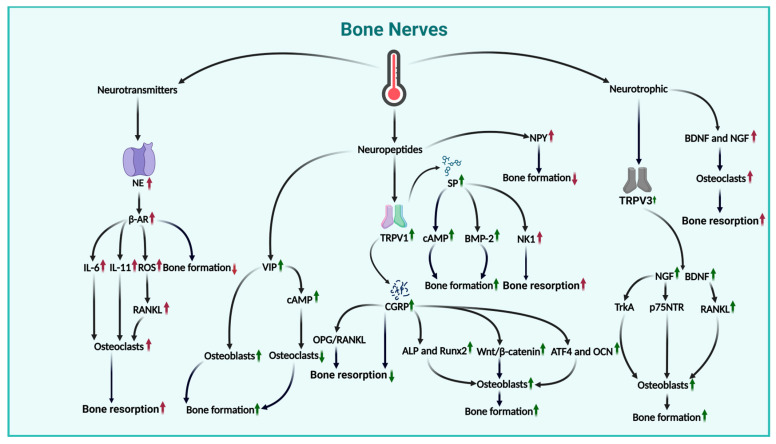
The multifaceted impact of the thermal environment on bone nerves. The affect of the thermal environment on bone nerves through encompassing neurotransmitters, neuropeptides, and neurotrophic factors. The thermal environment enhances bone resorption by upregulating NE in neurotransmitters and downregulates bone formation. It also upregulates bone formation by increasing VIP and CGRP in neuropeptides, but the upregulation of NPY downregulates bone formation. The effect of SP is relatively complex; it simultaneously upregulates both bone formation and bone resorption, similar to the effects produced by neurotrophic factors on the right side. (Green indicates a positive effect on bone health, red indicates a negative effect, an upward arrow indicates upregulation, and a downward arrow indicates downregulation).

In summary, heat stress from the thermal environment can release the NE neurotransmitter, which can stimulate bone resorption. Moreover, heat exposure can release the VIP and NPY neuropeptides and activate TPRV1 and thus release CGRP neuropeptides. In addition, SP, VIP, SP, and CGRP can promote bone formation through various pathways, whereas NPY can inhibit bone formation. Heat exposure can also release two neurotrophic factors, namely, NGF and BDNF, which can have a promoting effect on bone formation and bone resorption. Therefore, the impact of the thermal environment on bone nerves presents a complex regulatory pattern, with interactions and antagonisms between the factors. In conclusion, the mechanisms of the effect of the thermal environment on bone nerves require further in-depth research to fully understand the role of the thermal environment in regulating bone metabolism. (For detailed impacts of the above sections, please refer to Figure 6).

### 4.2. Effects of Thermal Environment on Bone Angiogenesis

Exposure of human primary osteoblasts and human outgrowth endothelial cells to mild heat stress (41 °C, one hour) twice daily for 14 consecutive days has been shown to increase the expression of HSP90, HSP70, and HSP27 [58]. HSP70 can promote angiogenesis by regulating BMP activity and IL-6 expression [120]. Meanwhile, HSP90 can promote angiogenesis and tubular structure formation by stabilizing matrix metalloproteinase-2 [121]. The thermal environment can upregulate VEGF, which is a key growth factor that can promote angiogenesis [58]. VEGF can induce endothelial cell proliferation and migration and thus promote new blood vessel formation. In addition, VEGF can regulate blood vessel growth and branching and modulate interactions between endothelial cells and the surrounding cells and hence maintain vascular stability and integrity. Such functions play a crucial role in maintaining normal bone vasculature functions [122]. Furthermore, fibroblast growth factors can promote bone angiogenesis. For instance, heat shock treatment (42 °C for 2 h, single exposure) has been observed to stimulate cells to release FGF-1, an effective inducer of new blood vessel formation in vivo [123].

**Figure 7:**
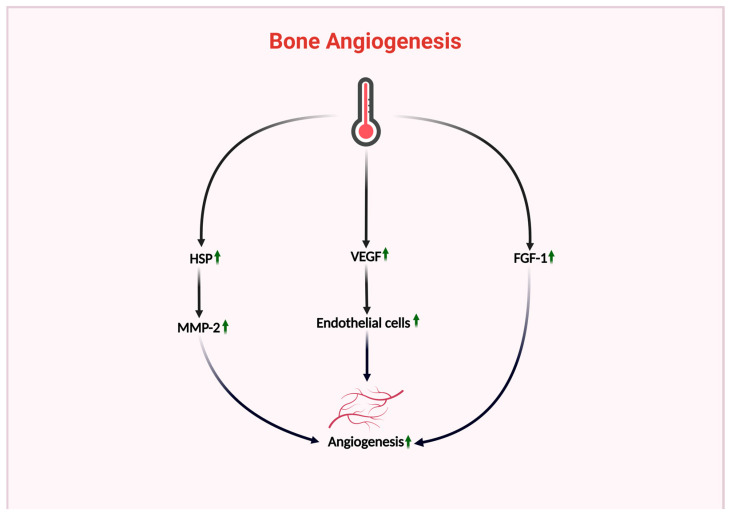
The affects of thermal environment on bone vasculature. The impact of thermal environment on bone vasculature, which positively promotes bone angiogenesis through upregulation of HSP, VEGF, and FGF-1. (Green indicates a positive effect on bone health, red indicates a negative effect, an upward arrow indicates upregulation, and a downward arrow indicates downregulation).

Therefore, the thermal environment can upregulate HSPs and promote the production of growth factors such as VEGF and FGF, which play a positive role in bone vasculature generation and function. (For detailed impacts of the above sections, please refer to Figure 7).

### 4.3. Effects of Thermal Environment on Bone Marrow Fat

**Figure 8:**
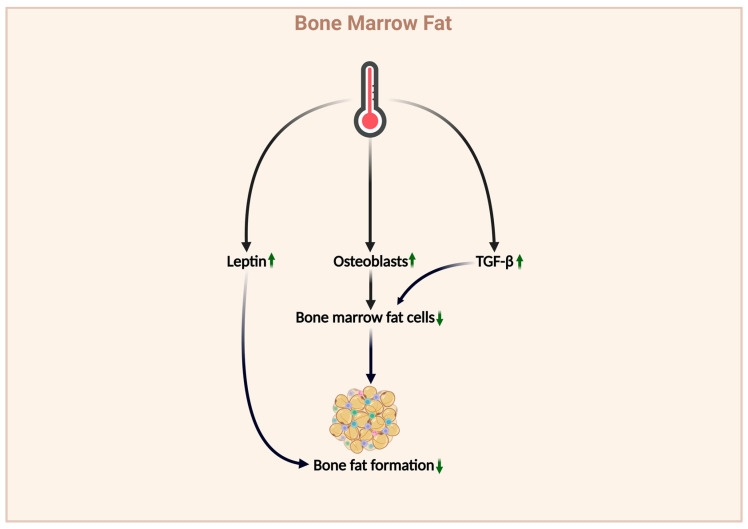
The affects of thermal environment on bone fat. The impact of thermal environment on bone fat, which upregulates leptin, enhances osteoblast activity, and reduces bone fat production through TGF-β. (Green indicates a positive effect on bone health, red indicates a negative effect, an upward arrow indicates upregulation, and a downward arrow indicates downregulation).

Bone marrow fat results from the accumulation of adipocytes within the bone marrow and serves as an energy store and plays an important role in the bone microenvironment [124]. Although research proving the effects of the thermal environment on BMF is lacking, existing potential mechanisms suggest a possible connection between the two factors. Bone formation and bone fat generation demonstrate balance, which is closely related to LEP. LEP deficiency can lead to decreased bone formation and increased bone fat [125]. As mentioned previously, the thermal environment can upregulate LEP expression. Bone marrow adipocytes and osteoblasts originate from BMSCs, and a competitive relationship exists between the two types of cells [124]. As mentioned previously, the thermal environment can promote the differentiation of BMSCs into osteoblasts. The findings suggest that the thermal environment may inhibit bone fat production [126], which is further supported by the behavior of TGF-β. The thermal environment can also promote TGF-β activation, and TGF-β has been proven to inhibit adipocyte generation. The two findings suggest that the thermal environment may inhibit BMF production through TGF-β. (For detailed impacts of the above sections, please refer to Figure 8, and for all the major experiments in this chapter, please see Table 3).

## 5. Methods

This review, adhering to SANRA (Supplementary Materials) guidelines [127], searched PubMed through January 2025 using keywords including “bone”, “bone metabolism”, “bone microenvironment”, “heat shock”, “heat exposure”, “heat stress”, “osteoblast”, “osteoclast”, “osteocyte”, “hot environment”, “neurofactor”, “thermal environment”, “hBMSCs”, “bone marrow fat”, “bone angiogenesis”, and “bone cells.” Inclusion criteria: peer-reviewed English articles quantifying heat exposure (e.g., ≥37 °C, duration, and frequency) and investigating direct mechanisms of thermal environments on the bone microenvironment (e.g., osteocyte activity, stem cell differentiation, angiogenesis, and bone metabolism). Both experimental and observational studies with extractable quantitative/qualitative data (e.g., bone density, gene expression) are included. Exclusion criteria: studies with non-quantified heat interventions (or insufficient experimental detail), no control group, and unavailable full-text grey literature. Screening involved abstract and full-text evaluation. Due to heterogeneity in experimental design, heat parameters, and outcome measurements, a meta-analysis was not feasible; findings are narratively synthesized to highlight thermal environment effects on bone.

## 6. Future Perspectives

Current research shows a complex relationship between temperature and bone health: in the context of climate warming, the lower incidence of fractures in low-latitude populations suggests that temperature may have a protective effect, but structural abnormalities such as cortical bone thinning occur simultaneously, requiring careful assessment of long-term temperature effects. From a clinical perspective, although mouse experiments indicate that thermal environments help improve bone loss, two limitations should be noted: first, at the molecular level, heat stimulation may simultaneously activate bone resorption pathways; second, significant physiological differences exist between mice and humans—mice experience more dramatic metabolic rate changes and have different thermoregulatory mechanisms, so extrapolation of experimental results to humans requires caution, particularly considering variables such as gender differences in basal body temperature [128]. Bone bioengineering is currently a hot topic that can be applied to fracture and osteoporosis treatment, and new biological scaffolds for osteoporosis treatment need to address both systemic metabolic abnormalities and local microenvironment changes, this means we also need to consider changes in the bone microenvironment caused by ambient temperature [129]. Bioactive scaffolds can promote bone repair by releasing factors such as IGF and VEGF, which can be activated by thermal environments, suggesting potential synergistic effects between heat stimulation and biological scaffolds [130].

In bone microenvironment research, the mechanism by which gut microbiota regulate bone metabolism has been elucidated previously, but the molecular-level regulatory pathways by which thermal environments affect gut microbiota have not been fully clarified [131]. Direct experimental evidence supporting bone vasculature and bone fat is still lacking. Future research needs to construct a multidimensional variable analysis system: in the experimental parameter dimension, a systematic examination of temperature gradients (intensity), exposure duration (acute/chronic), and intervention cycles (single/repeated) and their synergistic effects is needed; in the research subject dimension, standardized models encompassing species differences (rodents/primates), developmental stages (juvenile/adult/aging), and gender dimorphism (male/female) should be established. This will provide a unified paradigm for data integration and comparison among different research teams. In the future, as thermal environments’ effects on relevant molecular pathways are gradually elucidated, precision medical solutions based on environmental temperature interventions may revolutionize clinical pathways for osteoporosis and fracture repair. The synergistic application of this temperature-sensitive treatment concept with bone bioengineering technologies (such as bioactive scaffolds) may achieve dynamic optimization of bone defect microenvironments through a “temperature-material co-regulation” model, potentially demonstrating unique advantages in the combined treatment of bone healing disorders and metabolic bone diseases.

This paper currently has three limitations. First, there is a lack of effective translation mechanisms between animal experimental and human clinical trial data, limiting the transition of research findings to clinical applications. Second, temperature intervention parameters (such as intensity ranges from 27 to 400 °C, cycles varying from single exposure to continuous for one month, and duration lacking unified standards) have not yet established a standardized system, resulting in significant methodological heterogeneity among included studies. Finally, there is currently a lack of successfully translated cases for human clinical applications, constraining the practical application value of the research.

## 7. Conclusions

Thermal environments precisely regulate the skeletal system through a triple regulation of “structural morphology-cellular dynamics-molecular mechanisms”: At the tissue level, moderate heat exposure promotes bone density and longitudinal growth while improving fracture load and yield point, though potentially affecting geometric shape and thickness, whereas continuous high temperatures harm bone structure by reducing toughness and rigidity; at the cellular level, thermal environments directly influence osteoblast/osteoclast proliferation/apoptosis balance while regulating osteocyte network activity and bone marrow mesenchymal stem cell fate decisions, forming temperature-dependent metabolic regulatory circuits; at the molecular level, heat stress activates neural factors such as CGRP and NPY while coordinating angiogenesis and fat inhibition through VEGF and TGFβ.

However, current research has methodological limitations: Macrostructurally, studies rely primarily on mouse models with scarce human clinical evidence and unsystematic control of temperature parameters and biological variables. In cells, complex molecular regulatory mechanisms often lack sufficient validation against specific changes; molecularly, the bidirectional regulatory characteristics of neural factors on bone metabolism remain incompletely elucidated, while effects on bone angiogenesis and bone–fat relationships require additional experimental evidence.

## Figures and Tables

**Table 1:** The main experiments described in the chapter on the effects of thermal environment on the macroscopic structure of bones.

Study Design/Method	Species/Cell Type	Temperature	Observable Pros/Cons	Reference
Logistic regression, controlling for age, sex, vaccination status, sanitation, and wealth index	Children	<30 °C 30–35 °C ≥35 °C	Pros: promotes limb growth Cons: may stunt growth	[18]
Unilateral heat exposure on mice for 14 days; bone growth measured	C57BL/6 mice	40 °C 30 °C	Pros: increased femur/tibia length Cons: no effect on humerus	[19]
Daily 40 °C heat treatment on right limb for 40 min over 7 days; X-ray and OTC labeling measured bone length	C57BL/6 mice	40 °C 30 °C	Pros: measured bone length changes Cons: partial data used	[20]
16S rRNA, RNA sequencing, and microbiome transplantation	mice	34 °C	Pros: improved bone strength Cons: warmth affected food intake	[13]
Studied effects of cold, noise, and heat stress on bone cortical thickness	rats	10 °C 21.5 °C 33 °C	Cons: heat stress negatively impacted bone thickness	[29]
Randomized controlled trial with tibial loading and Raloxifene treatment	mice	22 °C 32 °C	Pros: improved bone structure Cons: thermoneutral caused weight loss	[30]
Heat exposure on pregnant rats; offspring skeletal malformations evaluated	rats	37 °C ≥42 °C 41 °C 42 °C	Cons: high temperature caused skeletal malformations	[34]
Heat shock on pregnant rats; embryos analyzed for protein synthesis	rats	37–38° 41 °C 42–42.5 °C	Cons: heat caused developmental abnormalities	[35]
Cell viability and mechanical function tests on heated femurs	rats	45–60 °C 50 °C to 90 °C	Cons: 60 °C killed bone cells	[39]
Randomized controlled trial with mice housed at 22 °C or 32 °C	mice	32 °C	Pros: 32 °C suppressed bone loss Cons: 22 °C caused bone loss	[49]
6-week sauna study on young men; bone density measured	Humans	100 ± 2 °C	Pros: increased bone density	[50]
Heat shock on hMSC-TERT cells; osteogenic differentiation assessed	hBMSC	41 °C 42.5 °C 44 °C	Pros: enhanced osteogenic differentiation Cons: long-term heat may have negative effects	[53]
Heat shock on hBMSCs and Mg-63 cells; proliferation and mineralization measured	hBMSC	33–45 °C	Pros: 39 °C and 41 °C enhanced mineralization Cons: 42.5 °C and 45 °C inhibited proliferation	[54]
Mild heat stress on osteoblasts and endothelial cells; angiogenesis and osteogenesis assessed	pOB OECs (Osteoblasts)	41 °C	Pros: enhanced angiogenesis and osteogenesis	[58]

**Table 2:** The main experiments in the chapter on the effects of thermal environment on bone cells.

Study Design/Method	Species/Cell Type	Temperature	Observable Pros/Cons	Reference
Sauna sessions (30 min) 3×/week; blood samples pre/post sauna; qRT-PCR for HSPA1A, HSPB1, IL6, and IL10 mRNA; and ANOVA	Humans	982 °C, 18 ± 2 °C	Pros: athletes showed better heat stress adaptation (↑IL10, ↓IL6 mRNA)	[66]
Lentiviral overexpression of HSPA1A in rBMSCs; qPCR, WB, IF for ALP, RUNX2, OCN, and COL1A1; ALP activity, ARS, micro-CT, and histology; DKK1 to validate Wnt/β-catenin; and cell sheet transplantation in rat fracture model	Rats	37 °C	Pros: HSPA1A overexpression enhanced osteogenic differentiation (↑ALP, RUNX2, OCN, COL1A1, and calcium deposition) via Wnt/β-catenin activation	[67]
Heat stress (42 °C for 0–60 min) on MSCs; WB, ICC; ANOVA, Newman–Keuls post-hoc analysis	Rats	42 °C 37 °C	Pros: heat stress significantly induced Hsp27 (48×) and Hsp70 (174×) expression, peaking at 48 h and returning to baseline by 120 h	[68]
Three lentiviral vectors tested for transduction efficiency in rat and human MSCs; cell passage, cryopreservation, species effects, and hypoxia/ischemia survival with HSP70 overexpression	Rats Humans	37 °C	Pros: HSP70 overexpression enhanced MSC survival under hypoxia/ischemia	[73]
HSP at 42 °C for 1 h on BMSCs; CCK-8, FC (Annexin V-FITC/PI), and WB for HSP70/HSP90	Rodents	42 °C	Pros: HSP reduced BMSC apoptosis, enhanced proliferation, upregulated HSP70/HSP90, and protected GCs from cisplatin-induced apoptosis	[76]
Long-term heat exposure (40 °C) on chondrocytes, osteoblasts, MC3T3E1, ROS 17/28; cell counting, FC, Rh123 staining, Coulter counter, and BCA	Rabbits, Mice, Humans	40 °C	Pros: chondrocytes showed increased proliferation at 40 °C Cons: osteoblasts, MC3T3E1, and ROS 17/28 cells had reduced proliferation and viability at 40 °C	[80]
Heat shock (33 °C to 45 °C) on hBMSCs and Mg-63; BrdU, crystal violet, ALP assay, and ARS staining	Humans	33 °C 39 °C 41 °C 425 °C 45 °C	Pros: 39 °C and 41 °C enhanced proliferation, ALP activity, and mineralization Cons: 425 °C and 45 °C inhibited proliferation; 33 °C had no significant effect	[54]
MC3T3-E1 exposed to 44 °C for 0–8 min; HSPs (HSP27, HSP47, HSP70), bone-related proteins (OPN, OCN, OPG, BSP, ALP, MMP-9), VEGF, and MTS assay	Mice	44 °C	Pros: heat stress alone or with GFs induced HSPs (especially HSP70), ↑OPG, and ↑VEGF Cons: heat stress inhibited MMP-9, potentially affecting bone remodeling	[81]
Heat stimulation (37 °C to 50 °C) on MG-63; TOPflash/FOPflash luciferase assay, PCR array for Wnt-related genes, and inhibitors (LY294002, rapamycin, U0126, and Dkk-1)	Humans	37 °C 448 °C 466 °C 475 °C	Pros: Heat activated Wnt signaling, ↑β-catenin nuclear accumulation, and ↑Wnt ligands (Wnt1, Wnt3a, Wnt8a, and Wnt10a) Cons: high temperatures (eg, 475 °C) may inhibit cells	[85]
Heat exposure (47 °C for 1 min) on MLO-Y4; fluorescence microscopy, FC, RT-PCR, and co-culture experiments	Mice	47 °C 37 °C	Pros: heat-treated cells influenced neighboring cells via secreted factors	[91]
RCT: 90 min cycling at 15 °C (CON) or 35 °C (HEAT); core temp (Trec), muscle temp (Tmus), HR, RPE, and blood samples (cytokines, hormones, metabolites, and WBC count)	Humans	15 °C 35 °C	Pros: HEAT group showed ↑Trec, ↑Tmus, and ↑stress hormones (adrenaline, noradrenaline)	[97]

**Table 3:** The main experiments in the chapter on the effects of thermal environment on the bone microenvironment.

Study Design/Method	Species/Cell Type	Temperature	Observable Pros/Cons	Reference
ELISA for CGRP, tested cannabinoids (anandamide, THC) on CGRP release	Mice (wild-type, CB1−/−, TRPV1−/−)	47 °C	Pros: TRPV1 activation linked to CGRP release Cons: TRPV1 knockout mice showed reduced heat-induced CGRP release	[101]
Micro-CT, histology (H&E), IHC, RT-qPCR, ELISA, WB, and BrdU assay	Rats (hBMSCs)	37 °C	Pros: CGRP inhibited TNF-α, reduced inflammation, regulated bone markers (RANKL, OPG, and OPN)	[102]
BMSCs isolation, osteogenic induction; FC, IHC, qPCR, WB, and Transwell assay	Rats (hBMSCs)	37 °C	Pros: SP ↑BMP-2, osteogenic genes (ALP, collagen I, osteocalcin, and RUNX2), VEGF, and migration; activated Wnt/β-catenin	[106]
BMSCs and BMMs culture; ALP activity, osteocalcin, and RANKL levels; WB, ELISA, TRAP staining, and immunofluorescence	Mice (hBMSCs, BMMs, RAW 2647)	37 °C	Pros: SP ↑ALP, Runx2, mineralization; ↑TRAP+ cells, bone resorption; and activated NF-κB	[107]
RT-qPCR for NPY and ASIP mRNA; ANOVA, Bonferroni test	Gallus gallus domesticus	40 °C 30 °C	Pros: acute heat stress ↑NPY expression	[109]
Immersion in 41 °C water, cycling in 41 °C environment; rectal temp, heart rate, and RIA for hormone levels	Humans	41 °C 37 °C 10 °C	Pros: heat load ↑PRL, β-endorphin, and VIP levels	[112]
Calcium imaging, IHC, WB, ELISA, qPCR, TRPV3 inhibitors, and behavioral analysis	Humans (keratinocytes) Mice (C57BL/6)	22–23 °C 33 °C 37 °C 39 °C 36–38 °C	Pros: TRPV3 ↑channel activity under heat stress	[114]
NGF treatment, inhibitors (LY294002, U1026), shRNA TrkA knockdown; migration, tube formation assays, and WB	Humans (chondrocytes HMVEC)	37 °C	Pros: NGF ↑FGF2 via PI3K/Akt and ERK/MAPK, promoting angiogenesis	[115]
Immersion in 42 °C or 35 °C water; core temp, MAP, HR, plasma cortisol, BDNF, S100b, and blood cell count; and repeated measures ANOVA	Humans	42 °C 35 °C	Pros: hot HOI ↑serum BDNF levels	[117]
Transwell co-culture, MTT assay, ALP staining, Alizarin red staining, ELISA for NGF/BDNF, RT-qPCR, ANOVA, and Newman–Keuls test	Rats (Schwann cells, osteoblasts)	37 °C	Pros: BDNF and NGF ↑osteoblast proliferation and differentiation	[118]
Cell culture (MM cells, BMSCs, and pre-OCs), WB, immunofluorescence, ELISA, co-culture, SCID-rab mouse model, and inhibitors (U0126, LY204002)	Humans (hBMSCs pre-OCs), Mice (SCID-rab)	37 °C	Pros: BDNF ↑RANKL via ERK; BDNF inhibition ↓RANKL, ↓osteoclast activity	[119]
Immunoprecipitation, WB, chemical crosslinking, cell proliferation, tube formation assays, Apoe−/− mouse model, and siRNA knockdown	Humans (HAECs), Mice (Apoe−/−)	37 °C	Pros: HSP70 ↑BMP-4 effects, mediated IL-6 pro-calcification	[120]

## Supplementary Materials

The following supporting information can be downloaded at: https://www.mdpi.com/article/10.3390/ijms26083501/s1.

## Abbreviations

activating transcription factor 4	ATF4
alkaline phosphatase	ALP
angiopoietin-1	Ang-1
angiopoietin-2	Ang-2
apoptotic protease activating factor 1	Apaf-1
bone marrow mesenchymal stem cells	BMSCs
bone mineral density	BMD
bone morphogenetic protein-2	BMP-2
brain-derived neurotrophic factor	BDNF
calcitonin gene-related peptide	CGRP
cysteine-aspartic protease-3	caspase-3
downregulating cathepsin K	Ctsk
Heat shock 70-kDa protein 1A	HSPA1A
heat shock protein 27	HSP27
heat shock protein 70	HSP70
heat shock protein 90	HSP90
heat shock proteins	HSPs
interleukin-11	IL-11
interleukin-6	IL-6
leptin	LEP
matrix metalloproteinase 9	Mmp9
nerve growth factor	NGF
neuropeptide Y	NPY
norepinephrine	NE
osteocalcin	Ocn
osteopontin	Opn
osteoprotegerin	Opg
p75 neurotrophin receptor	p75NTR
parathyroid hormone	PTH
phosphatase 5b	Trap5b
precursor of caspase-9	procaspase-9
receptor activator of nuclear factor kappa-B ligand	RANKL
Runt-related transcription factor 2	Runx2
second mitochondria-derived activator of caspase	Smac
substance P	SP
succinic dehydrogenase	SDH
transforming growth factor-β	TGF-β
transient receptor potential vanilloid 4	TRPV4
tropomyosin receptor kinase A	TrkA
tumor necrosis factor α	TNF-α
vascular endothelial growth factor	VEGF
vasoactive intestinal peptide	VIP
X-linked inhibitor of apoptosis protein	XIAP
β-adrenergic receptors	β-AR
